# PSNSleep: a self-supervised learning method for sleep staging based on Siamese networks with only positive sample pairs

**DOI:** 10.3389/fnins.2023.1167723

**Published:** 2023-06-06

**Authors:** Yuyang You, Shuohua Chang, Zhihong Yang, Qihang Sun

**Affiliations:** ^1^School of Automation, Beijing Institute of Technology, Beijing, China; ^2^Institute of Medicinal Plant Development, Chinese Academy of Medical Sciences and Peking Union Medical College, Beijing, China

**Keywords:** sleep staging, self-supervised learning, Siamese networks, contrastive learning, positive sample pairs

## Abstract

Traditional supervised learning methods require large quantities of labeled data. However, labeling sleep data according to polysomnography by well-trained sleep experts is a very tedious job. In the present day, the development of self-supervised learning methods is making significant progress in many fields. It is also possible to apply some of these methods to sleep staging. This is to remove the dependency on labeled data at the stage of representation extraction. Nevertheless, they often rely too much on negative samples for sample selection and construction. Therefore, we propose PSNSleep, a novel self-supervised learning method for sleep staging based on Siamese networks. The crucial step to the success of our method is to select appropriate data augmentations (the time shift block) to construct the positive sample pair. PSNSleep achieves satisfactory results without relying on any negative samples. We evaluate PSNSleep on Sleep-EDF and ISRUC-Sleep and achieve accuracy of 80.0% and 74.4%. The source code is publicly available at https://github.com/arthurxl/PSNSleep.

## 1. Introduction

Human sleep data are used in a variety of medical diagnoses, health care, and other applications (Wulff et al., 2010), and are commonly collected using polysomnography (PSG). It consists primarily of an electroencephalogram (EEG), an electrooculogram (EOG), an electromyogram (EMG), and an electrocardiogram (ECG). However, they are difficult to recognize and must be annotated by sleep specialists who have been well trained in the field. PSG data are always segmented into epochs of 30 s for analysis. In addition, the sleep stages of each individual are classified by experts according to sleep manuals such as the Rechtschaffen and Kales (R&K; Wolpert, 1969) and the American Academy of Sleep Medicine (AASM; Iber et al., 2007). There are a number of traditional deep learning approaches (Phan et al., 2018; Supratak and Guo, 2020; Zhu et al., 2020; Guillot and Thorey, 2021), which require a large amount of labeled data for training. It is very challenging to apply these methods to sleep classification when labeling these recordings is much more challenging than labeling an image (Mai and Yu, 2021).

The self-supervised learning method has attracted a lot of attention in recent years. It can be used to extract effective representations from unlabeled data and achieve similar performance to supervised learning with limited annotation information (Jing and Tian, 2019). Among various self-supervised learning methods, contrastive learning is favored by researchers because of its excellent performance (Lê Khắc et al., 2020). Oord et al. (2018) proposed Contrastive Predictive Coding (CPC). The negative samples were selected from the current batch and the entire model was trained using the loss function NCE (Gutmann and Hyvärinen, 2010) known as InfoNCE. As well as proving that self-supervised learning is universal in many different fields, they also proved that it has many advantages. As Wu et al. (2018) demonstrated, self-supervised learning can be achieved by maximizing the distinction between instances. They adopted a memory bank to store representations, which expanded the selection range of negative samples to the entire dataset. He et al. presented Momentum Contrast (MoCo; He et al., 2019), which was used for self-supervised visual representation learning. The updated strategy maximized consistency between negative samples and improved performance (Chen X. et al., 2020). However, the success of such methods depends greatly on the selection of negative samples in the training process (Jaiswal et al., 2020).

An alternative method of self-supervised learning is to learn invariant representations from different views of the original data (Zhou et al., 2021). As a means of achieving self-supervised learning, Siamese networks (Caron et al., 2020; Bardes et al., 2021) are used to maximize the similarity between the outputs (representations) of two branches of the Siamese networks. Chen T. et al. (2020) proposed SimCLR. It simplified contrastive self-supervised learning and did not rely on specific architectures or memory banks. A BYOL method has been proposed by Grill et al. (2020), and a SIMSIAM method has been proposed by Chen and He (2020). A prediction module is included in both of these methods, which introduces asymmetry into the original Siamese network. On the basis of previous research, Zbontar et al. proposed Barlow Twins (Zbontar et al., 2021), which incorporate redundancy reduction strategies. Assran et al. (2022) proposed Masked Siamese networks (MSN). It matched the representation of an image view containing randomly masked patches to the representation of the original unmasked image. A critical aspect of these methods is the composition of the data augmentations, which plays a crucial role in the results (Wang and Qi, 2021).

In recent years, some researchers have tried to apply self-supervised learning to sleep staging, hoping to free sleep experts from the tedious labeling work. SleepDPC was proposed by Xiao et al. (2021) and based on two dedicated learning principles, predictive and discriminative. It could discover underlying semantics from raw EEG signals. Cosleep is a representational learning framework that is based on a multi-view co-training mechanism that was proposed by Ye et al. (2022), along with a memory module that was added to the framework. Chang et al. (2022) proposed DSSNet, which combined the classical framework of DeepSleepNet (Supratak et al., 2017) and the classical self-supervised learning loss function InfoNCE. The TS-TCC was proposed by Eldele et al. (2021), used two different augmentations to get two views and adopted a contextual contrasting module to learn discriminative representations. The above methods have achieved good results. However, some of these studies rely too heavily on the selection of effective negative samples, and some of their network structures are overly complex.

In order to better apply self-supervised learning method to sleep staging, and free it from excessive dependence on negative samples, we propose PSNSleep. It can achieve better performance than other self-supervised methods by using Siamese networks and only a positive sample pair. The positive sample pair is constructed using a simple data augmentation method. Then, two CNNs and a GRU are used as branches of the Siamese network to extract general representations. A network’s overall training goal is to maximize the similarity between pairs of positive samples in order to increase its performance. In addition, we introduce asymmetry into the Siamese network and adopt different update strategies for the parameters of the two branches.

In summary, this paper is mainly devoted to developing a novel self-supervised learning method based on Siamese networks for sleep staging. In the representation extraction part of the Siamese network, we adopted two CNNs and a GRU. This network structure is more suitable for sleep data and can extract multi-view representations. At the same time, we introduced an asymmetric structure of a prediction in one branch of the Siamese network to prevent the occurrence of collapse solutions. An augmentation strategy designed to eliminate dependence on negative samples and create positive pairs. We introduced mixup and time shift augmentations. The mixup learns foreground information by mixing different background information. The time shift views adjacent sleep epochs as positive pairs. We evaluated our framework on two public datasets. The results show that our method is effective for sleep staging. Additionally, we conducted ablation experiments to explore the effects of different data augmentation methods.

## 2. Materials and methods

**Figure 1:**
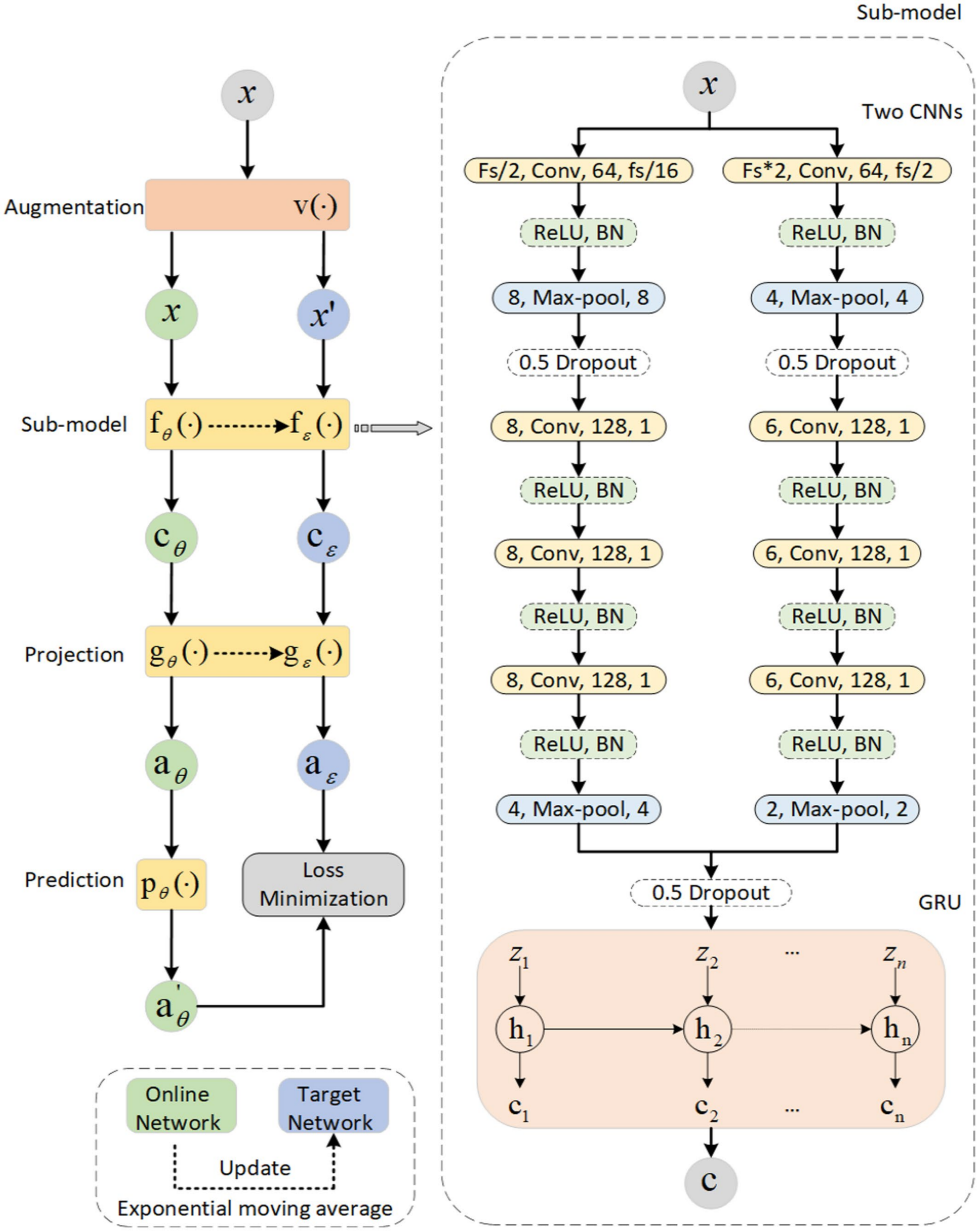
The overview architecture of our model. The left part of the figure is a Siamese network. The two branches are the online network and the target network. The parameters of the target network are dynamically updated according to the online network. The right part of the figure shows the structure of the sub-model, consisting of two CNNs and a GRU.

We propose a new self-supervised learning method to extract general representations from single-channel EEG without expecting to learn differences between the current sample and negative samples. The method is called PSNSleep. Figure 1 illustrates the architecture of PSNSleep.

### 2.1. Data augmentation blocks

#### 2.1.1. The time shift block

**Figure 2:**
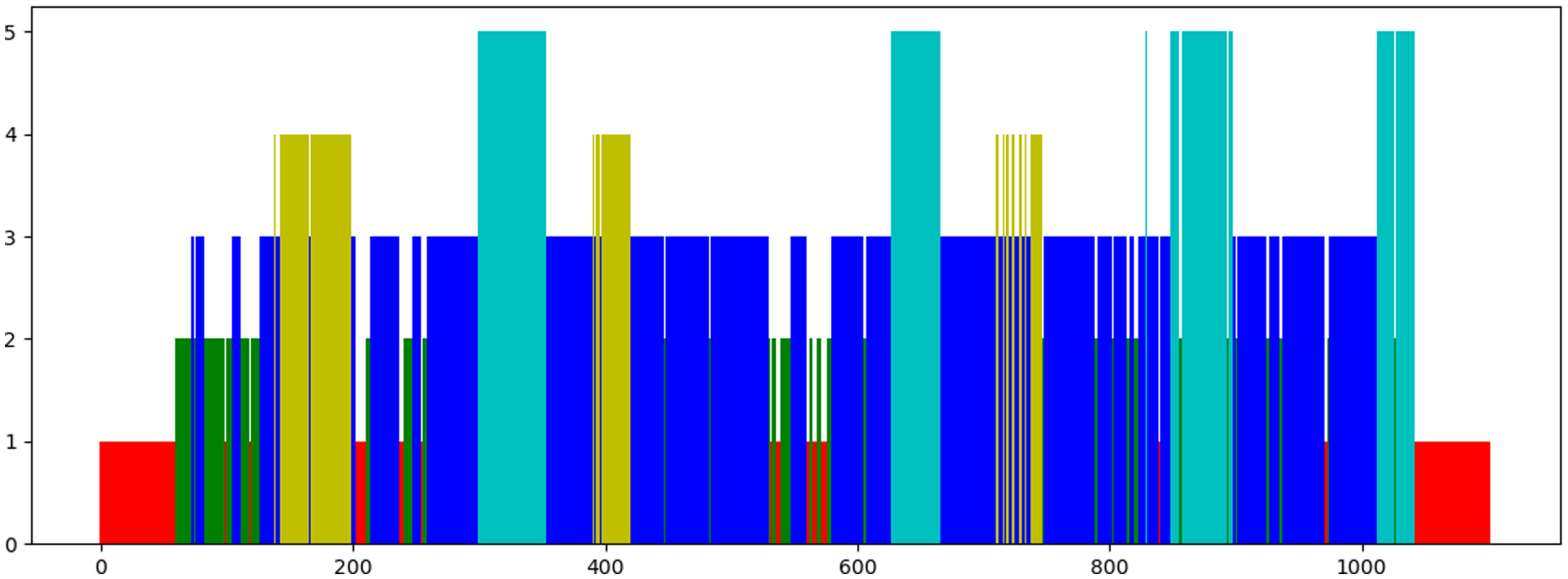
Sleep distribution map for a subject throughout the night. Stages of the same type have the same color and height. A *Y*-axis represents the classification of sleep, where 1 represents W, 2 represents N1, 3 represents N2, 4 represents N3, and 5 represents REM. During a period of time, the height and color are the same, indicating that they are in the same phase of sleep.

The sleep data are continuous, which means that sleep epochs are generally in the same stage for a period of time. A subject’s sleep data throughout a night is visualized in Figure 2; we can see the continuity between two adjacent sleep epochs. And the probability of adjacent two sleep epochs happen to be in the transition stage of sleep, which means they belong to two sleep stages, is very small. As a result, we may ignore this situation and consider two sleep epochs to be in the same sleep stage as a whole. After adopting this augmentation method, we obtained encouraging experimental results, indirectly demonstrating that ignoring this situation does not have much impact on our experiments. We can choose the adjacent sleep epochs at the same stage as a positive sample pair since their waveforms are similar, which indicates that they have a high degree of similarity. For the sleep signal at epoch t in the overnight sleep data, the adjacent epoch t+1 can be considered as its positive sample (Mai and Yu, 2021). It should be noted that the continuity of sleep stages is an assumption. For people with diseases, e.g., sleep apnea, narcolepsy, it might not hold due to the sleep fragmentation they suffer from.

#### 2.1.2. The mixup block and the Gaussian block

A batch of normalized EEG samples is used as input. In the mixup block, each sample is mixed with the other sample in a small ratio to obtain a mixed sample (Niizumi et al., 2021). It is also pertinent to mention that the other sample has been randomly selected from the current batch. The batch size is large enough to ensure the randomness that the sample selection is random. This operation can be viewed as changing the background information of a sample. In detail, the mixed sample adds background that is produced by the mixup block, while the raw sample has no background. The purpose of the above operations is to create a positive sample pair. They share most of the information, which we call foreground when referring to the area of audio recognition, but have different backgrounds. Therefore, in our method, the model can be improved by focusing on the foreground and ignoring different backgrounds in order to learn similar information between positive sample pairs. We actually require a representation of similar information extracted from the positive sample pair.

We adopt a basic mixup calculation method, and the calculation formula is,

(1) $${\tilde{x}}_{i}=\left(1-\gamma \right)x_{i}+\gamma x_{k}$$

where $x_{i}$ is the current sample, $x_{k}$ is the other sample that is used to mix, γ is the mixing ratio. Furthermore, a higher mixing ratio implies a greater proportion of background from the other sample. ${\tilde{x}}_{i}$ is the mixed sample. $x_{k}$ is randomly selected from the current batch.

For the Gaussian block, the principle is similar to the mixup block. The difference is that the mixed part is no longer other sleep samples, but rather the Gaussian noise we randomly generate. Considering that the signal about the Gaussian noise is generally small, it is directly superimposed on the original sleep data rather than mixed in a certain ratio. The current sample is superimposed with Gaussian noise generated at random, the calculation formula is,

(2) $${\tilde{x}}_{i}=x_{i}+g_{i}$$

where $x_{i}$ is the current sample and $g_{i}$ is the gaussian noise.

#### 2.1.3. The random mask block

Assuming that the length of an EEG sample is *N*, we can say that the sample consists of *N* patches. In order to maintain global information, we randomly select patches to mask. We set the values of the randomly selected patches to 0 to ensure that the raw data and the augmentation have the same length. For the waveform of sleep data, masking some points randomly does not have a significant impact on the overall trend of the waveform. In other words, the masked waveform contains similar graphic information compared to the original waveform. They have high similarity and can be considered as a positive sample pair. In the training process, our network is used to predict masked patches using the powerful learning capability of neural networks, which facilitates learning off-the-shelf representations more effectively (He et al., 2021).

#### 2.1.4. The scaling block

In addition to mixing with other signals to generate positive samples, we can also only rely on the sample itself to generate. We add random variations to the signal and scale up its magnitude. The specific realization is to scale the sleep data point by point by multiplying it with a random-number. The magnitude scaling can determine the similarity between positive sample pairs to a certain extent. The variation in amplitude can be expressed as follows,

(3) $${\tilde{r}}_{i}=\rho \times r_{i}$$

where $r_{i}$ is the magnitude of the current signal, ${\tilde{r}}_{i}$ is the scaling magnitude, and ρ is the scaling rate.

### 2.2. Siamese network

The Siamese network consists of two branches that are called online network and target network. Both of them share a similar architecture. The branch of online network has three parts: a sub-model that is used as a representation extractor, a projection, and a prediction. The prediction is absent from the other branch of the target network, which makes it a bit different. This self-supervised learning method is named PSNSleep.

#### 2.2.1. Representation extractor

The specific structure of the sub-model is shown in Figure 1. The model is composed of convolution layers and a recurrent layer. A convolution layer consists of two branches, one with a big-filter and the other with a small-filter, which allows time-frequency features to be extracted. In actuality, the recurrent layer is a gated recurrent unit (GRU), which is used to learn sequential epoch features. Taking the sub-model of the online network as an example, the process is as follows:

(4) $$f_{\theta }\left(\cdot \right)=f_{\left(CNN,\theta_{1}\right)}\left(\cdot \right)+f_{\left(GRU,\theta_{2}\right)}\left(\cdot \right)$$

(5) $$z_{t}=f_{\left(CNN,\theta_{1}\right)}\left(x_{t}\right)=\delta \left(w_{s}x_{t}+b_{s}\right)+\delta \left(w_{l}x_{t}+b_{l}\right)$$

(6) $$c_{t}=f_{\left(GRU,\theta_{2}\right)}\left(z_{t}\right)=GRU\left(z_{t},h\right)$$

Where $f_{\theta }\left(\cdot \right)$ represents the sub-model, $f_{\left(CNN,\theta_{1}\right)}\left(\cdot \right)$ and $f_{\left(GRU,\theta_{2}\right)}\left(\cdot \right)$ represent the CNNs and the GRU respectively, $z_{t}$ is the time-frequency feature, and $c_{t}$ is the final representation we need.

#### 2.2.2. Projection and prediction

It is very helpful to have a projection and a prediction. In previous studies, the projection has been shown to improve performance. In addition, the asymmetry of the two branches of the Siamese network caused by the prediction of the future can help the whole model learn more information and avoid collapsed solutions. Both are composed of two fully-connected layers, and all of the layers have 960 units. The projection also includes two additional operations, batch normalization (BN) and activation using rectified linear units (ReLU). The operations of these two parts are as follows:

(7) $$a_{\theta }=g_{\theta }\left(c_{\theta }\right),a_{\varepsilon }=g_{\epsilon }\left(c_{\epsilon }\right)$$

(8) $$a_{\theta }^{\prime }=p_{\theta }\left(a_{\theta }\right)$$

where $a_{\theta }.$ and $a_{\varepsilon }$ are the output of representations of c through the projection. $a_{\theta }^{\prime }$ is the output of the prediction, which is used to predict $a_{\varepsilon }$

#### 2.2.3. Update strategy of the Siamese network parameters

Although the online network and target network have many similarities, their update strategies are completely different. The parameters θ of the online network are constantly updated during the training of the whole model. In order for the online network to be trained, the regression targets are provided by the target network. The parameters ε in this model are exponential moving averages of the θ . After each training step, we perform the following updates:

(9) θ=optimizer(θ,∇L,η)

(10) ϵ=τϵ+(1−τ)θ

where ∇L is the gradient of loss function L , η is the learning rate of optimizer, and τ is the target decay rate.

#### 2.2.4. Loss function

We duplicate a raw single-channel EEG (which has been normalized) into two copies. One does not require any processing, and the other is processed to get $x^{\prime }$ through a data augmentation module. We put x and $x^{\prime }$ into two branches of the Siamese network, online network and target network, to get the outputs $a_{\theta }^{\prime }$ and $a_{\epsilon }$ respectively. In addition, L2-normalization is applied as well. To measure the similarity of the positive sample pair, we calculate the mean squared error between the normalized prediction and target projection.

(11) $$\bar{a_{\theta }^{\prime }}=\frac{a_{\theta }^{\prime }}{∥a_{\theta }^{\prime }{∥}_{2}},\bar{a_{\epsilon }}=\frac{a_{\epsilon }}{∥a_{\epsilon }{∥}_{2}}$$

(12) $$L_{\theta ,\epsilon }=\bar{∥a_{\theta }^{\prime }}-\bar{a_{\epsilon }}{∥}_{2}^{2}=2-2\cdot \leq \frac{\langle a_{\theta }^{\prime },a_{\epsilon }\rangle }{∥a_{\theta }^{\prime }{∥}_{2}\cdot a_{\epsilon }{∥}_{2}}$$

We symmetricize the loss $L_{\theta ,\epsilon }$ by separately feeding $x^{\prime }$ to the online network and x to the target network to compute $L_{\theta ,\epsilon }^{\prime }$ . The loss function can be defined as follows:

(13) $$L=\left(L_{\theta ,\epsilon }+L_{\theta ,\epsilon }^{\prime }\right)/2$$

During each training step, a stochastic optimization step is performed to minimize the loss L.

### 2.3. Experiments

We evaluate our self-supervised learning method by using single-channel EEG signals from two public datasets: Sleep-EDF (Goldberger et al., 2000; Kemp et al., 2000) and ISRUC-Sleep (Khalighi et al., 2016).

#### 2.3.1. Sleep-EDF

It was an excellent dataset for the study on sleep staging in aging. The data were obtained in a 1987–1991 study of age effects on sleep in healthy Caucasians aged 25–101. The SC cohort of the Sleep-EDF contains 20 healthy subjects. Each PSG recording has two EEG signal channels, Fpz-Cz and Pz-Cz. All of them have the same sampling rate of 100 Hz. According to R&K standards, all recordings are categorized into eight categories (W, N1, N2, N3, N4, REM, MOVEMENT, and UNKNOWN). Data need to be preprocessed in accordance with the AASM standard. N3 and N4 are combined into N3, MOVEMENT, and UNKNOWN are the start and end of the recording, respectively. It is only the Fpz-Cz channel that we use.

#### 2.3.2. ISRUC-sleep

As a test of the fit of our model to a generalized situation, we adopted this dataset to test the performance of the model. It contains 100 subjects. Each PSG recording has six EEG signals with the same 200 Hz sampling rate. All recordings are segmented into 30-s epochs and visually scored by two different sleep experts according to the guidelines of AASM, with the stages: W, N1, N2, N3, and REM. We only use the F3-A2 channel.

In Table 1, the numbers of 30-s EEG epochs for the five stages are presented. We employ zero-mean normalization to improve the speed of convergence of our model. The input data x is normalized to $\tilde{x}=\frac{x-\mu }{\sigma }$ , where μ is the average and σ is the standard deviation.

**Table 1:** The numbers of 30-s EEG epochs.

Dataset	W	N1	N2	N3	REM	Total
Sleep-EDF	8,285	2,804	17,799	5,703	7,717	42,308
ISRUC-Sleep	20,098	11,062	27,511	17,251	11,265	87,187

The input size of Sleep-EDF is (batch-size, 1, 3,000) and ISRUC-Sleep is (batch-size, 1, 6,000). Other basic settings include a batch size of 32, a model trained for 200 epochs, and a random seed of 2022. The optimizer we chose is Adam with a learning rate of 0.0001. A decay rate of 0.1 is set for the target network. For the augmentation block, the mixing ratio γ is configured to 0.4. In order to mine the sequence information between extracted representations, we add a simple sequence network before classification, which is composed of a GRU with 64 units. The sequence length is 5. Then a linear classifier is used to evaluate the performance of representations. It consists of two fully connected layers with 960 and 64 units, respectively. We use the sample without any data augmentation as the input of the sub-model from the online network. In the process of evaluation, we adopt its output as a representation. All parameters of the sub-model are frozen when we train the classifier. The optimization is performed using an Adam optimizer with a learning rate of 0.0001. We have also set the number of training epochs to 200. The cross-entropy loss function is minimized by training the classifier.

We adopted 10-fold cross-validation. Datasets are divided into 10 parts. In each fold, nine parts are used in the training process to help update the parameters of the Siamese network and the left part is used to evaluate. A confusion matrix is adopted to clearly show the classification result of each class. We use overall accuracy ( acc ), macro-averaging F1-score ( MF1 ), and Cohen’s Kappa coefficient ( κ ) (Sokolova and Lapalme, 2009) to measure the performance of our network specifically. They can be calculated as follows:

(14) $$acc=\frac{\sum_{c=1}^{C}TP_{c}}{N}$$

(15) $$MF1=\frac{\sum_{c=1}^{C}F1_{c}}{C}$$

where $TP_{c}$ presents the true positive samples when the class is c, $F1_{c}$ presents F1-score when the class is c, C presents the number of sleep stages, and N presents the total number of epochs.

## 3. Results

Table 2 shows the results of the previous methods compared with our model, including one supervised learning method and three self-supervised learning methods. We have developed a model that has a similar structure to DeepSleepNet at the representation learning stage. Both SleepDPC and Cosleep contain multi-channel EEG signals, while we only use a single-channel EEG signal. Besides, SleepDPC only used the SC cohort portion of the dataset during the experiment. The previous model DSSNet used a single-channel EEG signal and all data, but it relied too heavily on the selection of negative samples.

**Table 2:** The results of previous methods and PSNSleep.

Method	Sleep-EDF			ISRUC-Sleep		
	acc	MF1	*k*	acc	MF1	*k*
DeepsleepNet	0.820	0.769	0.76	-	-	-
SleepDPC	0.701	0.640	-	0.536	0.489	-
Cosleep	0.716	0.558	-	0.579	0.501	-
DSSNet	0.800	0.700	-	0.714	0.663	-
PSNSleep	0.808	0.738	0.737	0.744	0.710	0.668

For each fold, we use the last epoch’s result as the current fold’s result. Then, we can calculate the results of all folds to obtain the final performance metrics, as shown in Tables 3, 4. The accuracy of our method is 80.8% on Sleep-EDF and 74.4% on ISRUC-Sleep. It achieves the highest results among self-supervised methods. Our model improves the accuracy of Sleep-EDF and ISRUC-Sleep by 0.8% and 3%, respectively, compared to DSSNet. The accuracy distance between our self-supervised model and the classic supervised learning model DeepSleepNet is further reduced to 1.2%, on the dataset Sleep-EDF. In the validation experiment, as the experimental sleep data includes a certain proportion of aging PSG data, our model is capable of performing both non-aging and aging PSG data sleep staging tasks. Table 4 shows that our self-supervised learning method is applicable to sleep datasets and has achieved state-of-the-art performance. The gap between our method and the traditional supervised learning method is further shortened. PSNSleep representations have high generalization. At the same time, the results also prove that over dependence on negative samples is not necessary in self-supervised learning, and only using positive sample pairs can also achieve positive encouraging performance.

**Table 3:** Performance metrics on Sleep-EDF.

Predicted						Per-class metrics		
	W	N1	N2	N3	REM	PR	RC	F1
W	6,912	445	136	28	213	80.23	89.37	84.55
N1	585	993	566	15	625	35.98	35.67	35.82
N2	603	500	15,192	598	777	87.15	85.98	86.56
N3	157	5	672	4,856	6	88.27	85.25	86.73
REM	358	817	867	4	5,670	77.77	73.48	75.56

**Table 4:** Performance metrics on ISRUC-Sleep.

Predicted						Per-class metrics		
	W	N1	N2	N3	REM	PR	RC	F1
W	17,582	1,354	415	28	484	83.11	88.52	85.73
N1	2085	4,422	2,805	50	1,532	47.46	40.59	43.76
N2	869	1943	20,853	1802	1834	73.09	76.38	74.70
N3	74	40	3,194	13,697	190	87.79	79.66	83.53
REM	546	1,558	1,263	25	7,595	65.28	69.13	67.15

To explore how data augmentation methods affect representation performance, we conduct the ablation experiment in this section. In order to maximize the reliability of the comparable results, all experimental settings except the augmentation blocks have been kept the same. As a direct reflection of the performance of different data augmentation methods, we ignore sequence information in order to reflect the performance of the representations. The representations extracted by the self-supervised method are directly fed into the classifier. Our laboratory uses Sleep-EDF to conduct this ablation experiment.

**Figure 3:**
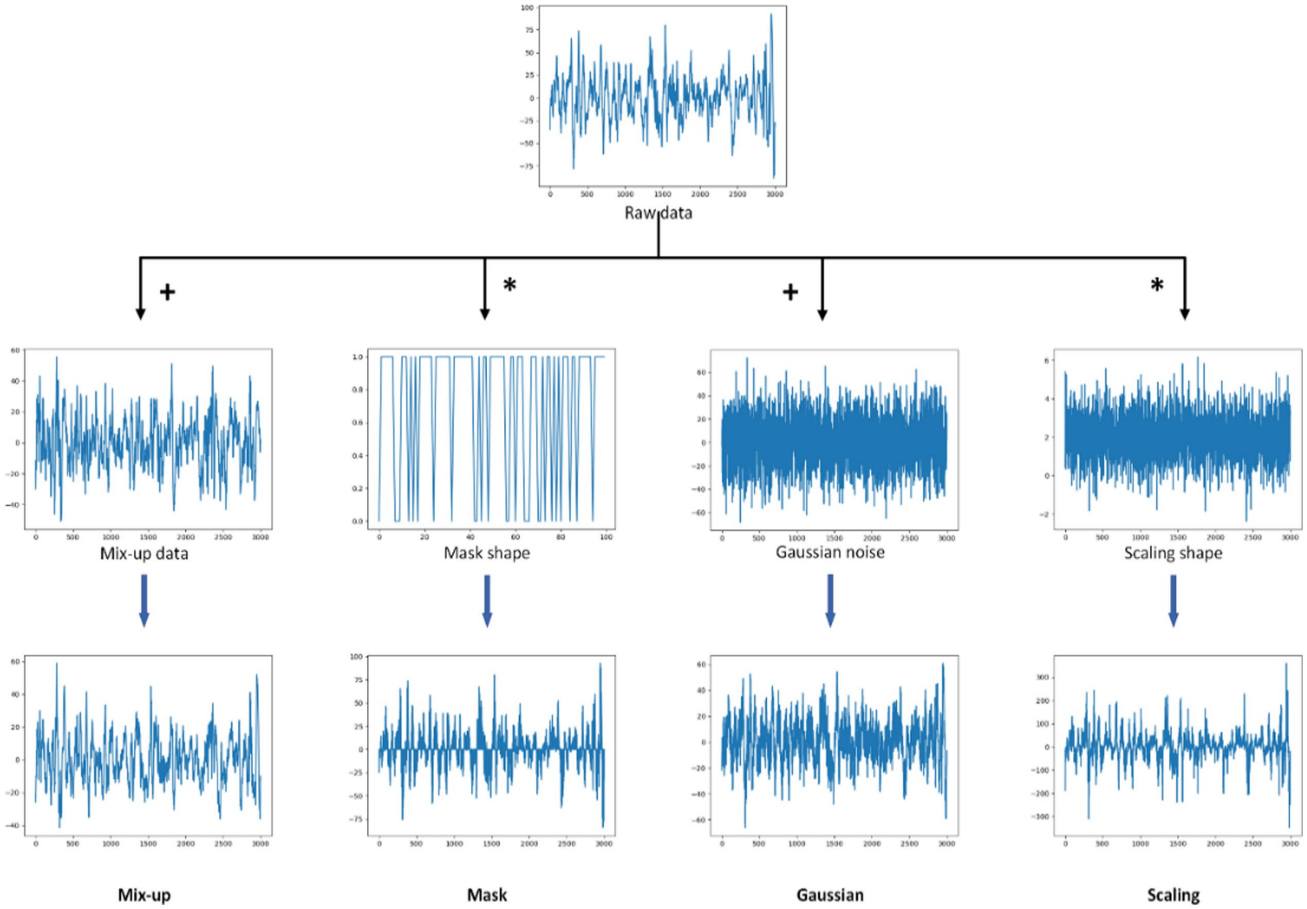
Diagram of different data augmentations. From left to right, the methods are mixup, mask, Gaussian, and scaling. The first row is the raw data, the second row is the augmentation data, and the last row is the result graphs. “^+^” represents the weighted sum of raw data and argumentation data in a certain proportion. “^*^” refers to the multiplication of elements one by one. A mask shape displays only the first 100 elements in order to facilitate viewing.

We tested six augmentation blocks: none, Gaussian, scaling, random mask, mixup, and time shift. Their specific operations are shown in Figure 3. The number of masked patches of the random mask is set to 100 (mask100) and 1,000 (mask1000) respectively. The results are shown in Table 5. The accuracy is 50.2%, 52.0%, 53.5%, 54.2%, 55.0%, 80.2%, and 80.5%, respectively. All data augmentation blocks have positive influences on the classification results compared with no augmentation. And the smaller number of mask patches may improve performance to a certain extent. For single augmentation, the time shift block and the mixup block have better results and their accuracy is over 80%.

**Table 5:** The performance of different data augmentations.

Augmentation	Acc	MF1	*k*
None	0.502	0.356	0.234
Gaussian	0.520	0.383	0.279
Scaling	0.535	0.388	0.320
mask1000	0.542	0.419	0.327
mask100	0.550	0.429	0.334
Mixup	0.802	0.710	0.728
Time shift	0.805	0.720	0.731

**Figure 4:**
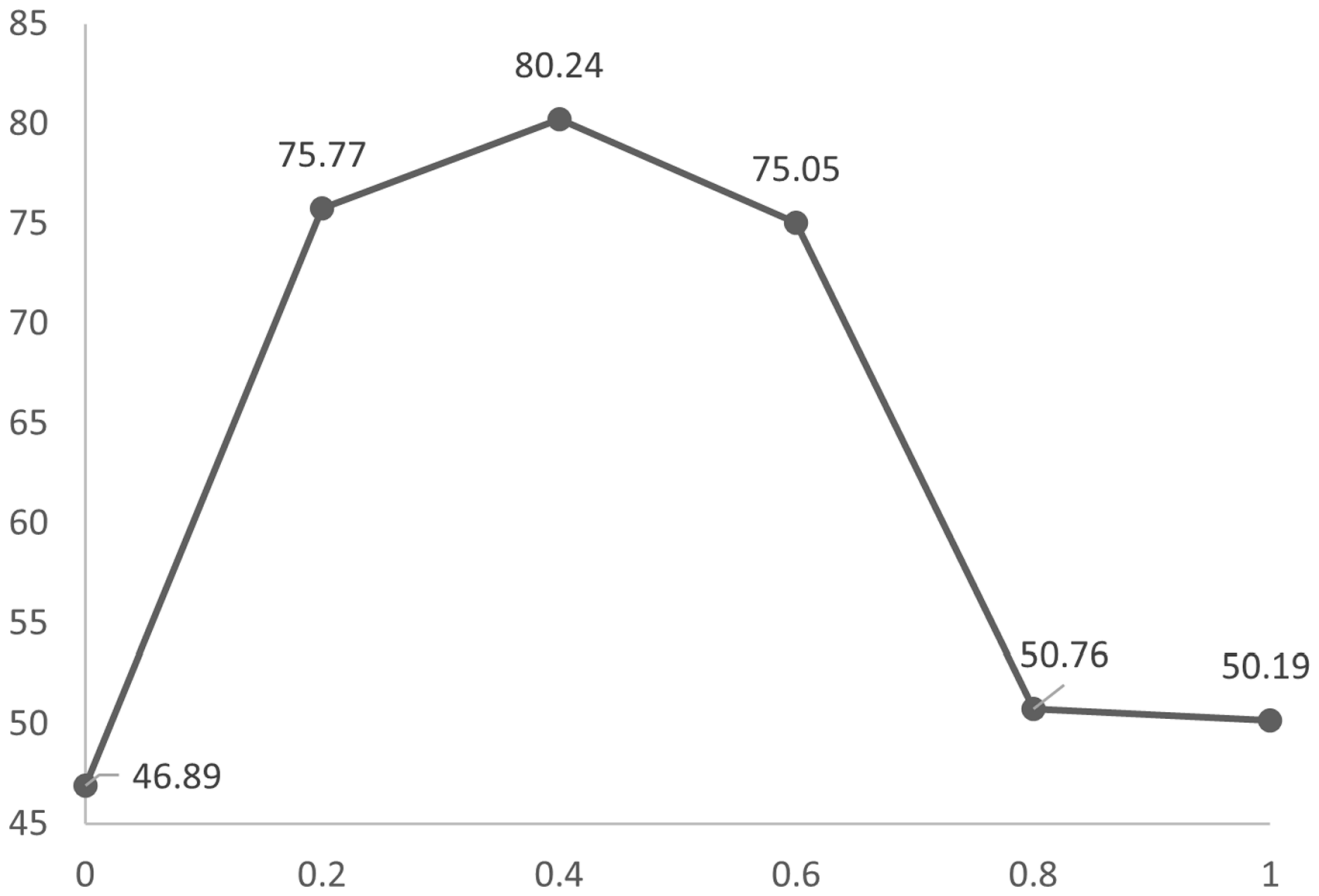
Accuracy at different mixing ratios is obtained by 10-fold cross-validation on the Sleep-EDF. The *Y*-axis represents accuracy and the *X*-axis represents the mixing ratio.

To explore the sensitivity of our model to the mixing ratio, we varied it to 0, 0.2, 0.4, 0.6, 0.8, and 1.0, respectively. The results are shown in Figure 4. Our model achieves the highest performance when it is set to 0.4. It is also essential that the mixing ratio is chosen appropriately. Too large or too small a mixing ratio will lead to a decline in results.

There are still some limitations. (1) Through ablation experiments, we selected the time shift block as our data augmentation method. However, sleep data continuity in this method is an assumption. For people with diseases such as sleep apnea, narcolepsy, it might not hold due to the sleep fragmentation they suffer from. (2) For comparison with previous studies, we selected F3-A2 and Fpz-Pz channels when the EEG derivations recommended by the AASM are F4-M1, C4-M1, and O2-M1. (3) We selected two datasets to validate the feasibility of our proposed method. However, the effectiveness of joint training and testing on two or more datasets remains to be verified. (4) In addition, information about open-source datasets is limited. We cannot determine whether data leakage occurs during the experimental process.

In the subsequent research: (1) We will explore whether using EEG signals from different channels of the same dataset impacts experimental performance. (2) We will conduct transfer learning on sleep data, which means training on one dataset and testing on another dataset. (3) Cesari et al. (2021) developed an automatic method to model sleep as a continuous and dynamic process and this method predicted aging more accurately. In our future work, we can combine it with our method. Specifically, the self-supervised learning method replaces the manual feature extraction process, aiming to provide features with better generalization performance for the subsequent classification process.

## 4. Conclusion

In this paper, we propose a novel self-supervised learning method called PSNSleep. It extracts representations from unlabeled EEG signals and achieves the highest performance of self-supervised learning methods. It overcomes the disadvantage that the performance of self-supervised learning depends largely on negative samples in sleep staging. Our architecture consists of a Siamese network with two CNNs and a GRU for representation extraction. A projection component based on previous experience is also included in our model. The use of prediction also facilitates the introduction of asymmetry and improves the performance of our network. The positive pair is constructed from data augmentations, which are essentially different views of the same sample. Data argumentation is one of the keys to ensuring that the method we propose is successful as well. We explored a variety of data augmentation techniques during the course of these experiments. The results show that the time shift block achieves the highest performance. The objective of our model is for the representation of the positive sample pair, which is made up of two branches of the Siamese network, to be highly similar. Our experimental results show that sleep staging based on self-supervised learning can also achieve competitive results when using only positive sample pairs.

## Data availability statement

The original contributions presented in the study are included in the article/supplementary material, further inquiries can be directed to the corresponding author.

## Author contributions

YY, SC, and ZY: conceptualization and methodology. YY, SC, and QS: software. YY: validation, data curation, supervision, and project administration. ZY: formal analysis and visualization. QS: investigation. YY and ZY: resources, writing—review and editing, and funding acquisition. SC: writing—original draft preparation. All authors contributed to the article and approved the submitted version.

## Funding

This study was supported by the National Natural Science Foundation of China (Nos. 81973744 and 81473579), CAMS Innovation Fund for Medical Science (CIFMS; Nos. 2022-I2M-1-018 and 2022-I2M-2-001), and the Beijing Natural Science Foundation (No. 7173267).

## Conflict of interest

The authors declare that the research was conducted in the absence of any commercial or financial relationships that could be construed as a potential conflict of interest.

## Publisher’s note

All claims expressed in this article are solely those of the authors and do not necessarily represent those of their affiliated organizations, or those of the publisher, the editors and the reviewers. Any product that may be evaluated in this article, or claim that may be made by its manufacturer, is not guaranteed or endorsed by the publisher.
